# MSC-EVs transferring mitochondria and related components: A new hope for the treatment of kidney disease

**DOI:** 10.3389/fimmu.2022.978571

**Published:** 2022-09-29

**Authors:** Jueyi Mao, Cuifang Li, Feifeng Wu, Zhou She, Senlin Luo, Xiaoyu Chen, Chuan Wen, Jidong Tian

**Affiliations:** Department of Pediatrics, The Second Xiangya Hospital, Central South University, Changsha, China

**Keywords:** mitochondria, renal diseases, treatment, extracellular vesicle (EV), immune

## Abstract

Kidney disease is a serious hazard to human health. Acute or chronic renal disease will have a significant negative impact on the body’s metabolism. The involvement of mitochondria in renal illness has received a lot of interest as research on kidney disease has advanced. Extracellular vesicles are gaining popularity as a means of intercellular communication in recent years. They have a close connection to both the nephropathy process and the intercellular transfer of mitochondria. The goal of this review is to present the extracellular vesicle transport mitochondria and its related biologically active molecules as new therapeutic options for the treatment of clinical kidney disease. This review focuses on the extracellular vesicles through the transfer of mitochondria and its related bioactive molecules, which affect mitochondrial energy metabolism, take part in immune regulation, and secrete outside the body.

## Background

The mitochondria are important cellular organelles, and their internal oxidation system delivers energy to cells *via* a series of metabolic events. In recent years, an increasing number of studies have revealed that mitochondria play a role not only in energy metabolism but also in immunological modulation and kidney disease by diverse pathways. Extracellular vesicles (EVs) have recently received considerable interest as a type of intercellular communication. Several studies have shown that EVs can transport mitochondria and its related bioactive molecule and create positive therapeutic effects. Hence, transporting technologies are likely novel therapeutic strategies for clinical kidney disease. This review will explore the role of the mitochondria in the histopathology of kidney disease and the therapeutic potential of EVs by regulating mitochondrial function. (Especially in kidney disease).

## The function of mitochondria

### The energy metabolic function of mitochondria

Mitochondria are double-membraned, elliptical organelles that consist of an outer membrane, inner membrane, stroma, and intermembrane space. The inner membranes are highly folded that form the “cristae.” These cristae contain respiratory chain-related enzyme protein complex, which is the main site of energy production *via* aerobic respiration in the mitochondria. As the key organelles of energy metabolism, mitochondria are constantly dividing and fusing. Mitochondrial division is mediated by the binding of cytoplasmic dynamicity-associated protein to the receptor mitochondrial fission protein 1 (Fis1) on the outer membrane of the mitochondria. Mitochondria reproduce by division or reduce their number by autophagy, which is how mitochondrial quality is controlled within the cell. Optic atrophy protein-1 (OPA1) on the inner membrane of mitochondria and mitochondrial fusion proteins (Mfn1 and Mfn2) on the outer membrane mediate mitochondrial fusion. Mitochondrial abnormalities can be weakened by fusing their contents, thereby allowing them to deal with greater energy demands during cellular stress (1). This process of continuous division and fusion is called mitochondrial dynamics, in which cells eliminate malfunctioning mitochondria, repair damaged mitochondria, relocate mitochondria to guarantee enough energy supply, and respond to environmental changes in a timely manner. Mitochondrial dynamics primarily manifests itself in cells by changing the morphology of the cristae to affect cell function—fused mitochondria have more cristae area and are more closely connected than divided mitochondria, thereby ensuring the patency of the electron transport chain and allowing for better use of the oxidative phosphorylation reaction. Divided mitochondria prefer to use glycolysis for energy metabolism. According to studies, preventing excessive mitochondrial division and boosting mitochondrial fusion improves cell stress tolerance (2).

### The immune-related function of mitochondria

Mitochondria play a role in immunological modulation through a variety of pathways in addition to energy metabolism.

(1) Immune modulation is aided by intermediates of the tricarboxylic acid cycle.

The tricarboxylic acid cycle is a vital physiological process for mitochondrial productivity, and it also plays a role in immune modulation. Citriconic acid, an intermediate product of the tricarboxylic acid cycle, has been shown to be able to manufacture fatty acids, nitric oxide, prostaglandin (3), and other compounds in M1-type macrophages (4), indicating that it can continue to synthesize anti-inflammatory chemicals in macrophages (5). Citriconic acid can also produce itaconic acid to inhibit isocitrate lyase and prevent the formation of tuberculosis bacilli and other microorganisms in the body (6).

(2) Immune function is influenced by mitochondrial dynamics.

Mitochondrial dynamics alter immune function by affecting mitochondrial location and consequently cellular energy supply. Microtubules and microfilaments hold mitochondria in place in cells, while motor driver proteins reside on the microfilaments, which can degrade the driver proteins in different directions to allow mitochondria to travel around in the cell (7). Mitochondria use this principle to travel to the synaptic location during T-cell activation, boosting the availability of ATP at the synapse and further controlling the active transport of calcium ions, causing T cells to become activated (8, 9). During activation, mitochondria in naive T cells tend to divide, causing the electron transport chain to become disjointed, increasing the formation of reactive oxygen species (ROS), which is beneficial to the activation of naive T cells (10). In effector T cells, mitochondria tend to divide for glycolysis, whereas memory T cells use mitochondrial fusion, which aids in memory T- cell long-term survival.

(3) Mitochondrial DNA plays a role in immunological regulation.

Because of its similarities to bacterial DNA, mitochondrial DNA (mtDNA) is also implicated in immunological control. In a variety of inflammatory models, mtDNA leakage has been detected. Although it is unclear how mtDNA is extracted from mitochondria, it is apparent that mtDNA plays a major role in immunity as damage-associated molecular patterns (DAMPs) (11). It is squeezed into the cytoplasm, where it is promptly identified by many DNA pattern receptors (including DAI, IFI16, DDX41, DNA-PK, and cGAS (12)) and causes a robust innate immune response (13). Furthermore, during apoptosis, mitochondria produce oxidized mitochondrial DNA, which binds to the NLRP3 inflammasome and enhances downstream apoptotic protease activity-1 activation and the generation of interleukin-1 (14). This way, mitochondrial DNA establishes a link between mitochondrial damage and apoptotic protease-1 (15).

(4) Mitochondria plays a vital role in the production of ROS. Mitochondrial ROS (mtROS) is mainly produced by electron transport chain (ETC) during the process of oxidative phosphorylation. The ETC contains four complexes, and some of it are H^+^ producing hydrotransmitters (complexes I, III, and IV). When oxygen radicals gain electrons before the expected reaction site (i.e., complex IV), they are converted into ROS (including peroxide, superoxide, and hydroxyl radical) (16). Therefore, complex I and complex III are the main ROS-producing sites (17). In addition, mitochondria generate ROS *via* pathways other than the respiratory chain (18). Uncoupling protein 2 (UCP2), for example, is a negative regulator of ROS in the mitochondria, and its mechanism needs to be investigated further (19). MtROS is also a key regulator of adaptive immunity. It has been discovered that mtROS can aid T-cell activation and contribute to the production of IL-2 (20). Furthermore, inhibiting T-cell glycerol-3-phosphate dehydrogenase-2 (GPD2), another enzyme that generates mtROS, reduces T-cell IL-2 production (21). According to the findings, mtROS may be directly or indirectly involved in the body’s immune process.

## The role of mitochondria in kidney disease

**Figure 1:**
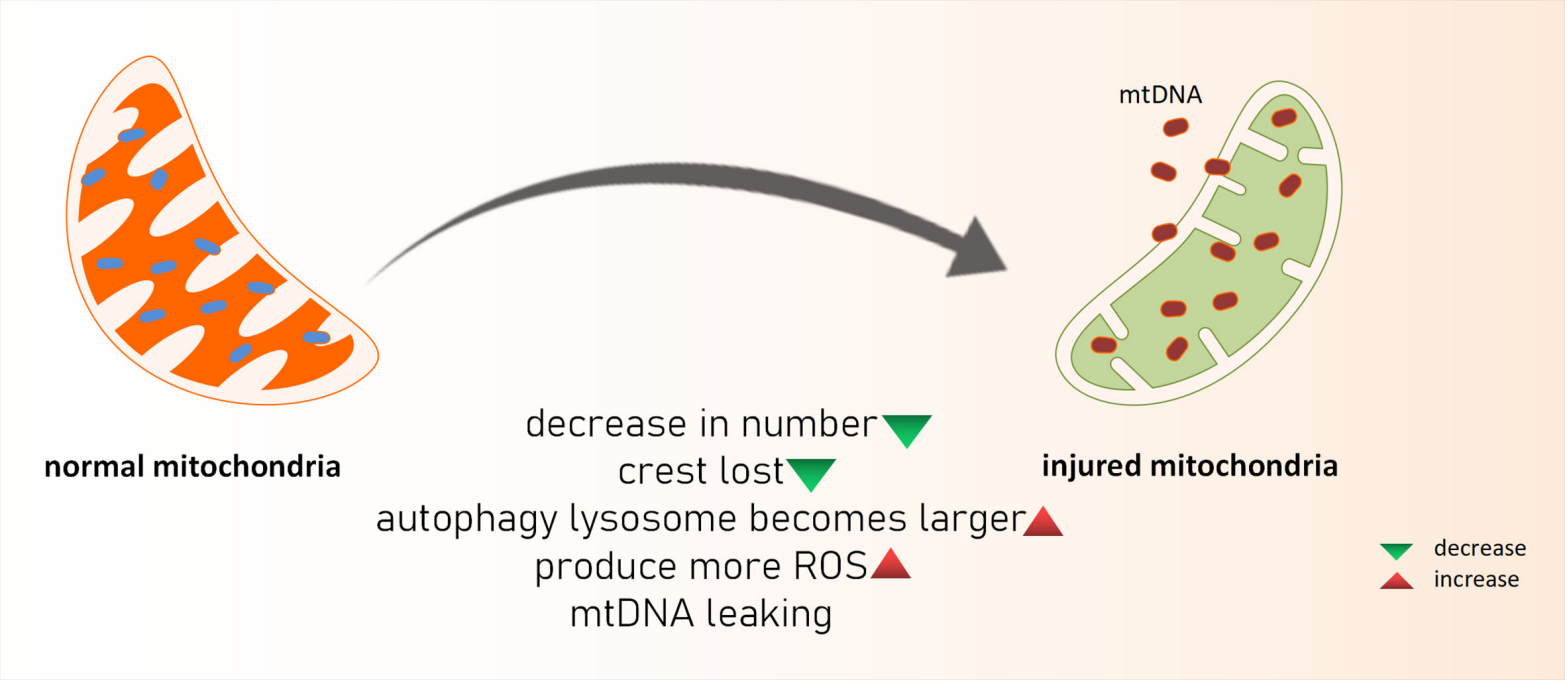
What happened to damaged mitochondria. mtDNA, mitochondria DNA; ROS, reactive oxygen species.

Acute kidney injury (AKI) is the sudden loss of renal function because of a variety of reasons. When AKI develops clinically, the glomerular filtration rate and urine volume both decline dramatically. Although the etiology of AKI caused by renal ischemia-reperfusion damage (IRI), nephrotoxic medications, sepsis, and other etiologies differs, they are all linked to oxidative stress, apoptosis, and the inflammatory response in renal tubular epithelial cells. AKI is also a key factor in the development of chronic renal disease (CKD) (22). As previously said, ROS is produced in mitochondria, and when a sufficient amount of ROS is produced that cannot be neutralized by the antioxidant system, oxidative stress occurs, and ROS can directly or indirectly damage nucleic acids, proteins, and lipids in cells. Excessive ROS products harm the biofilm, reducing its fluidity and increasing permeability, resulting in functional abnormalities in the biofilm (such as cell membrane rupture, mitochondrial membrane swelling and dissolution, and lysosomal membrane dissolution and rupture) as well as cell apoptosis (**Figure 1**). Hemodynamic alterations and renal fibrosis can be caused by oxidative stress that can exacerbate the onset and progression of kidney disease (23). The high oxidation activity of kidney mitochondria makes the organ vulnerable to oxidative stress damage, which can lead to kidney disease progression and renal failure (24). Excess ROS products serve as intracellular second messengers in the pathological process of AKI, releasing a variety of chemokines and inflammatory factors such as through multiple cellular inflammatory signal transduction pathways, causing the activation and proliferation of inflammatory cells, as well as migration to the injured site, thereby promoting the inflammatory response of the kidney, and ultimately leading to disease. In the mouse AKI model of sepsis induced by cecal ligation perforation, reduced oxidative stress is aided by inhibiting mitochondrial superoxide generation, which improves renal function and survival. Some researchers created a septic renal injury model in male Wistar rats and discovered that endotoxins and inflammatory mediators such tumor necrosis factor, interferon 7, and interleukin 1 produced *in vivo* can cause apoptosis and accelerate renal injury by increasing ROS levels (25). Furthermore, the ischemia-reperfusion mouse model revealed the opening of the mitochondrial permeability transition pore (mPTP) and subsequent cell death. Cyclophilin D (CypD) is a crucial element of mPTP, and the pp53 CypD complex has been linked to cell death. *In vitro* tests confirmed that the Pp53 CypD complex alters mitochondrial activity by mPTP opening, resulting in renal tubular cell death (10). This further highlights the importance of mitochondrial dysfunction in acute renal illness is the pathological process of AKI will continue to play a crucial part in chronic kidney injury. When AKI occurs, renal tubule shrinkage is observed, and the number of mitochondria in cells is considerably reduced, cristae are lost, autophagy-lysosomes are increased. At the same time, the oxidation-sensitive mitochondrial protein MPV17L was lost in rat kidney tissues, as was the expression of hypoxia markers and the content of glycolytic products like lactic acid and pyruvate, implying that mitochondrial depletion after AKI leads to irreversible metabolism, promoting the development of early fibrosis (26). In mice nephrectomy models, ROS has been shown to elevate inflammatory factors such as growth transforming factor (TGF-β), fibronectin (FN), and type IV collagen, resulting in chronic kidney injury and reduced renal function. Additionally, mitochondrial failure can activate the inflammasome, resulting in an inflammatory response (27). Damaged mitochondria release immune-related factors such as ROS, DNA, and cardiolipin, which further mediate the inflammatory activation of NLPR3 and the release of interleukin-18 (IL-18) and interleukin-1β (IL-1). It took a month after ischemia for these inflammatory factors to show a sustained increase in expression (28). Mitochondrial DNA interacts with endoplasmic reticulum molecules in a way known as *organelle cross-talk*, as a new inflammatory pathway, and it directly triggers tubulo-inflammatory responses and promotes the progression of CKD (29).

Mitochondria also play a role in inflammation. NETosis is a cell death mechanism that results in the formation of neutrophil extracellular traps (NETs), which are important in the host’s defense against bacterial infection (30). In recent years, it has been discovered that NETosis abnormalities and NETs clearance defects promote the production and release of type I interferon in systemic lupus erythematosus (SLE), based on the findings of related studies (31). It is linked to vascular complications as well as multiple organ injury (32). This research emphasizes the critical role of mitochondrial ROS in immune complex-mediated NETosis and the importance of mitochondria in the pathogenesis of SLE (33). At the same time, circulating cardiolipin of extracellular mitochondrial origin was discovered, which could explain why anticardiolipin autoantibodies are found in SLE or in the blood of patients with antiphospholipid syndrome (34).

## Role of extracellular vesicle transfer mitochondria and related bioactive molecules in disease

Extracellular vesicles have been found to have anti-inflammatory, anti-apoptotic, angiogenesis, and anti-fibrosis effects on kidney disorders, according to a number of recent research. Studies have also revealed that extracellular vesicles transport mitochondria and that these bioactive molecules have beneficial therapeutic effects on a variety of disorders, including those of the liver, lungs, central nervous system, and other organs. The kidney is an extremely energy-dependent organ, and mitochondrial dysfunction is intimately associated with the development of many renal disorders. Therefore, by transporting mitochondria and their associated components, extracellular vesicles’ therapeutic effect on the kidney can be achieved.

### MSC-EVs for treatment of renal disease

According to related reports, vesicles have been proven to have significant therapeutic effects in kidney diseases.

#### (1) Anti-inflammatory properties of EVs derived from stem cells have been discovered

In a mouse ischemia-reperfusion AKI model, stem-cell–derived vesicles reduced TLR2 and CX3CL1 expression in renal tubular cells and transferred related miRNAs to damaged renal tubular epithelial cells, reducing the infiltration of NK cells into renal damaged tissue and further reducing the inflammatory response (35).

#### (2) EVs derived from stem cells have the ability to promote proliferation while suppressing apoptosis

To achieve an anti-apoptotic effect, stem cell-derived EVs obtained from urine can protect podocytes, inhibit renal tubular epithelial cell apoptosis, inhibit the overexpression of Caspase-3 (a terminal splicing enzyme in apoptosis), and promote the proliferation of glomerular endothelial cells in a rat model of type I diabetes. EVs also contain growth factors such as TGF-β1, angiopoietin, and bone morphogenetic protein 7, which can help kidney cells survive (36).

#### (3) The active molecules in EVs can improve ischemic injury and promote endothelial cell regeneration, and EVs derived from stem cells can promote angiogenesis

ApoA1 released by EVs has been shown to reduce renal ischemic injury, neutrophil activation, and aggregation by inhibiting the effects of ICAM-1 and P-selectin on endothelial cells, resulting in improved renal injury (37).

#### (4) The anti-fibrosis effect of EVs derived from stem cells has been discovered

The release of mSC-derived, mirNA-LET7C-expressing EVs into the kidney inhibited TGF-1-induced expression of pro-fibrosis genes in target cells, alleviating renal fibrosis in a mouse model of unilateral ureteral occlusion (UUO) (38).

### Mesenchymal Stem cell-derived extracellular vesicles function by transporting mitochondria and their related active substances to target cells

**Figure 2:**
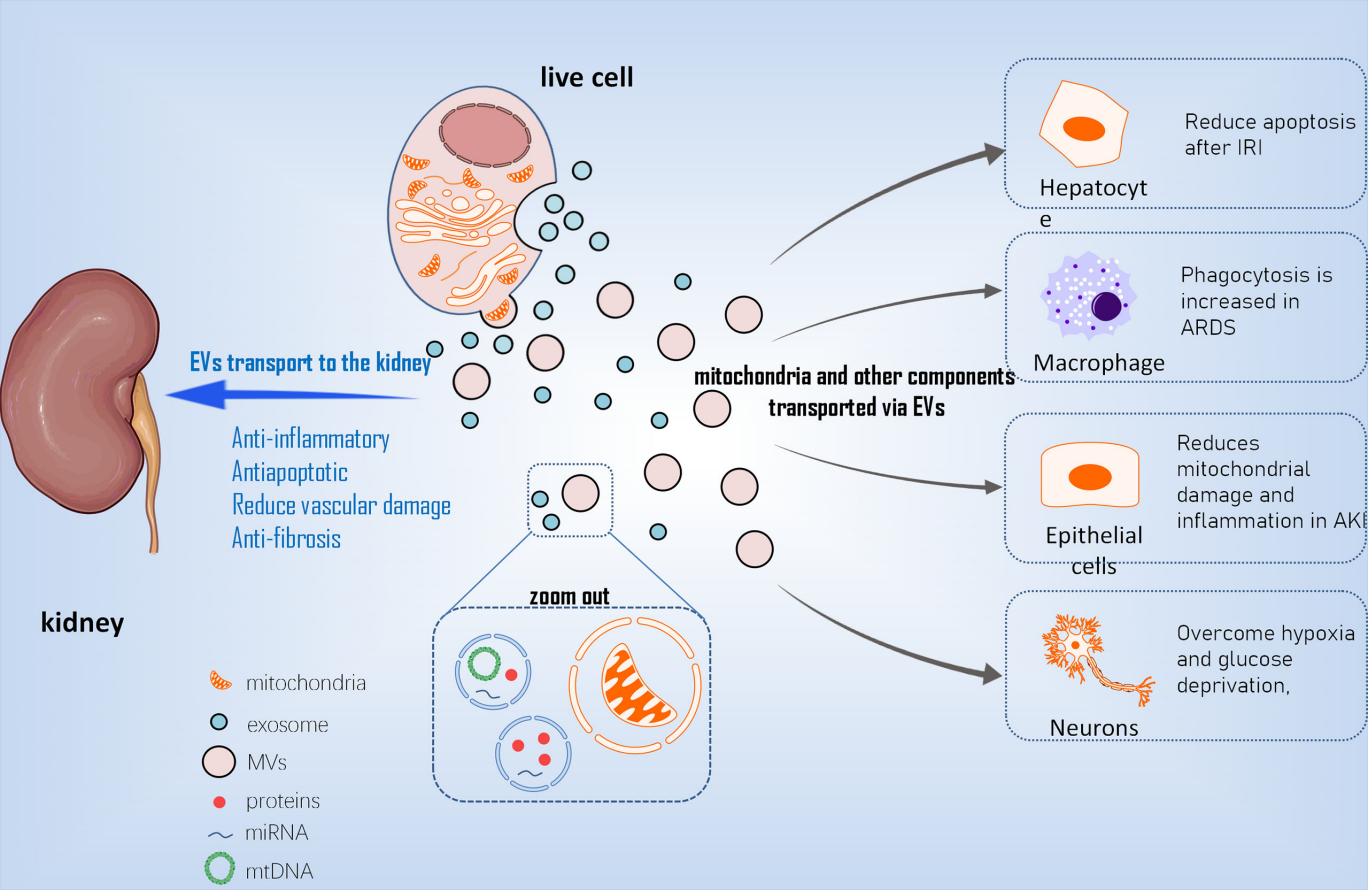
The mechanism of EVs transporting mt into different cells. EVs, extracellular vesicles; mt, mitochondria; MVs, Microvesicles; IRI, ischemical reperfusion injury; ARDS, acute respiratry distress syndrome; AKI, acute kidney injury.

Compared to stem cell therapy, stem cell derived EVs for cell repair have low immunogenicity, are easy to preserve and transport, and reduce other risks associated with stem cell therapy. It has been reported that stem cells can transport mitochondria to other cells using microvesicles (MVs) ranging in diameter from 0.1 m to 1 µm (39) (**Figure 2**). Other mitochondrial components, such as soluble proteins, the mitochondrial inner membrane, and mtDNA, are transported by exosomes with smaller particle sizes. EVs transport mediated by arrestin domain containing protein 1 acquires suitable mitochondria in bone marrow mesenchymal stem cells, and these tiny vesicles are eventually engulfed by macrophages (40). MSC-derived mitochondria in the cytoplasm are packaged into vesicles containing the autophagy marker light chain 3, which then migrate to the cell’s periphery and merge with outgoing germinating vesicles in the plasma membrane during transport. In the central nervous system, this EV-dependent mitochondrial transport pathway has also been observed between astrocytes and neurons (41). Stem cells restore damaged cell functions by secreting extracellular vesicles that transport mitochondria and other components. This goal is primarily accomplished in two ways (42). One method is to process depolarized mtDNA through vesicles to complete mitochondrial quality control (43). The second is that healthy cells secrete mitochondria and other components to help damaged cells recover their bioenergetics (44). Although there is a lot of indirect evidence that EVs play a role in the intercellular transport of mitochondria and their components, more direct evidence about the biological activity of MV-transported mitochondria is needed.

### Effects of Mesenchymal Stem cell derived-extracellular vesicles transfer of mitochondria and its related bioactive molecule on diseases

To begin with, EVs have unrivaled advantages in disease diagnosis. EVs can overcome the limitations of traditional sampling methods given their wide sources and availability in various body fluids, and many relevant studies have confirmed their diagnostic value. EVs containing mitochondria, for example, can be extracted from the blood of patients with systemic lupus erythematosus, and the concentration of these vesicles is linked to disease activity and inflammatory factor levels. Other research has found that EVs containing mitochondrial proteins can be extracted from the plasma of melanoma patients. These proteins are unique and distinguish this subpopulation of EVs from other cells, allowing for melanoma diagnosis with greater precision (45).

With the advancement of EVs research in recent years, EVs, particularly EVs derived from MSC, have been used in an increasing number of studies on diseases involving mitochondrial damage (1). MSC-EVS improved the effects of liver IRI in the ischemia-reperfusion model of hepatocytes by reducing intracellular ROS levels, alleviating hepatocyte apoptosis after ischemia-reperfusion, and preventing the formation of local Net by transferring functional mitochondria to intrahepatic neutrophils and restoring their mitochondrial function (46) (2).MSCs regulate human macrophages in the inflammatory environment of acute respiratory distress syndrome (ARDS) to reduce the production of pro-inflammatory cytokines, increase the expression of M2 phenotypic marker CD206, and improve macrophage phagocytosis. This effect is achieved by mitochondria-carrying MSC-EVS promoting macrophage oxidative phosphorylation to reveal a new mechanism of macrophage polarization regulation. Furthermore, this research suggests that mSC-EVS-induced changes in macrophages are sufficient to induce lung injury protection *in vivo* (47) (3). mSC-EVS has been shown to reduce mtDNA damage and inflammation after AKI in an ischemia-reperfusion–induced AKI model, which is dependent on the mitochondrial transcription factor A (TFAM) pathway. Transcription inhibition had no effect on the transfer of mitochondrial TFAMmRNA in recipient cells *via* EVs. As a result, MSC-EVS restored TFAM protein and TFAM-MTDNA complex (nucleoid) stability, reversing mtDNA deletion and mitochondrial oxidative phosphorylation (OXPHOS) defects in damaged renal tubular cells. As a result, MSC-EVS can effectively reduce mitochondrial damage and inflammation in cell and animal renal injury models by restoring TFAM expression and preventing mtDNA damage and cytoplasmic mtDNA leakage (48). In regenerative medicine, this study shows that MSC-EVS is a promising nanotherapy for attenuating various diseases characterized by mitochondrial damage (4). After a stroke, astrocyte-derived extracellular vesicles have been shown to transfer mitochondria to neurons *via* the CD83 signaling mechanism, allowing cells to survive in a state of relative hypoxia and glucose deficiency (41).

Extracellular vesicles are an important mode of communication between cells, and their transport mitochondrial bioactive molecule can play a role in immunomodulation either directly or indirectly. Platelets have been shown to secrete vesicles containing mitochondria, which are then broken down by phospholipase A2-IIA in neutrophils, releasing mtDNA, lysophospholipids, and fatty acids, which further activate neutrophils and promote their pro-inflammatory response (49). The ability of extracellular vesicles to participate in immune regulation is further demonstrated in this study.

## Conclusions

The kidney is a highly energy-dependent organ due to the need for reabsorption, and mitochondria have high oxidative activity and are the main site of ROS production. Furthermore, it is linked to the occurrence and progression of several kidney diseases (50). Extracellular vesicles have been shown to have the function of transferring mitochondria and their associated components to repair damaged cells and participate in immune regulation (shown in **Table 1**), which is a novel concept with significant benefits. Kidney diseases are caused by wide range of factors, all of which are linked to immune function. As previously stated, mitochondria play a role in the occurrence and progression of kidney disease through a variety of mechanisms, including ROS production, immune regulation, and energy metabolism. Extracellular vesicles have been shown in recent studies to have anti-inflammatory, anti-apoptotic, angiogenesis-promoting, and anti-fibrosis effects in kidney diseases. Meanwhile, research has shown that mitochondrial extracellular vesicle transfer has a positive therapeutic effect on diseases of the liver, lungs, central nervous system, and other organs. Therefore, do mitochondrial associated EVs transfer play a role in kidney disease, and can it be used as a new kidney disease diagnosis and treatment strategy? We believe that if it is used in the diagnosis and treatment of varied kidney diseases, we can explore the possibility of improved disease diagnosis and treatment, which can benefit more patients with acute and chronic kidney disease. The mechanism of MSC-EVS transfer to mitochondria and related bioactive substances still have great therapeutic potential in kidney diseases, which is worthy of more peers to participate in the study. However, the majority of the evidence supporting MV transfer to mitochondria is indirect, and whether the transferred mitochondria have comparable biological activity is also worth discussing. To conclude, EVs transport of mitochondria and its related components is very promising, and it deserves more peer participation in this field.

**Table 1:** Role of extracellular vesicles in a variety of pathoophysiological process.

Origin of Evs		Contents	Mechanism	Effect	Reference
Role of MSC-EVs in renal pathophysiology					
stem cells		miRNA	TLR2 and CX3CL1 expression in renal tubular cells were reduced, and NK cell infiltration to renal damage was reduced.	reduces inflammation in the kidneys	(35)
Urine-drived stem cells		Vegf,Ang,etc.	protect podocytes, inhibit the apoptosis of renal tubular epithelial cells and inhibit the overexpression of Caspase3	anti apoptosis in renal tubules	(36)
stem cells		ApoA1 Active substance	inhibition of ICAM-1 and P-selection on endothelial cells can reduce renal ischemia injury and neutrophil activation and aggregation	ameliorate kidney damage	(37)
mesenchymal stem cells		miRNA-let7c -	TGF-β1-induced expression of pro-fibrosis genes in target cells was inhibited	reduce renal fibrosis	(38)
Therapeutic effects of stem cell-derived mitochondria associated vesicles in disease					
human mesenchymal stem cells		Normal mitochondria	reduce ROS levels in damaged cells	reduce liver IRI	(46)
mesenchymal stem cells		mitochondria	promoted oxidative phosphorylation of macrophages and increased the expression of M2 phenotypic marker CD206 in macrophages	enhance the phagocytosis of macrophages	(47)
mesenchymal stem cells		TFAM	the stability of TFAM-mRNA complex and TFAM protein expression of mitochondria in damaged cells were restored, and mtDNA damage and leakage of target cells were prevented	reduce renal IRI	(48)
Astrocytes		mitochondria	CD83 mediated mitochondrial transfer to neurons	helps neurons survive lack of oxygen and glucose	(41)

ROS, reactive oxygen species; IRI, ischemical reperfusion injury.

## Author contributions

JM: Preparing the table and figures. Drafting the manuscript. CW and JT: Drafting the manuscript. Reviewing and editing the figures and table. Conceptualization and revising. CL, ZS, and FW: Reviewing and editing the manuscript. XC: Preparing the figure and table. All authors contributed to the article and approved the submitted version.

## Funding

This work was supported by the [National Natural Science Foundation of China #1] under Grant [number 82070758]; and [Hunan Provincial Key R&D Program Project#2] under Grant [number 2020SK2084].

## Conflict of interest

The authors declare that the research was conducted in the absence of any commercial or financial relationships that could be construed as a potential conflict of interest.

## Publisher’s note

All claims expressed in this article are solely those of the authors and do not necessarily represent those of their affiliated organizations, or those of the publisher, the editors and the reviewers. Any product that may be evaluated in this article, or claim that may be made by its manufacturer, is not guaranteed or endorsed by the publisher.
